# Isoquercitrin Ameliorates Cisplatin-Induced Nephrotoxicity *Via* the Inhibition of Apoptosis, Inﬂammation, and Oxidative Stress

**DOI:** 10.3389/fphar.2020.599416

**Published:** 2020-12-18

**Authors:** Hao Wang, Weiwei Xia, Guangfeng Long, Zhiyin Pei, Yuanyuan Li, Mengying Wu, Qian Wang, Yue Zhang, Zhanjun Jia, Hongbing Chen

**Affiliations:** ^1^ Department of Clinical Laboratory, Children’s Hospital of Nanjing Medical University, Nanjing, China; ^2^ Jiangsu Key Laboratory of Pediatrics, Nanjing Medical University, Nanjing, China; ^3^ Nanjing Key Laboratory of Pediatrics, Children’s Hospital of Nanjing Medical University, Nanjing, China; ^4^ Department of Nephrology, Children’s Hospital of Nanjing Medical University, Nanjing, China

**Keywords:** isoquercitrin, cisplatin, nephrotoxicity, apoptosis, inflammation, oxidative stress

## Abstract

Cisplatin is extensively used and is highly effective in clinical oncology; nevertheless, nephrotoxicity has severely limited its widespread utility. Isoquercitrin (IQC), a natural flavonoid widely found in herbage, is well known and recognized for its antioxidant, anti-inflammatory, and anti-apoptotic properties. However, the potential effects and mechanism of IQC in cisplatin-induced acute kidney diseases remain unknown. In this study, we postulated the potential effects and mechanism of IQC upon cisplatin exposure *in vivo* and *in vitro*. For the *in vivo* study, C57BL/6J mice were pretreated with IQC or saline (50 mg/kg/day) by gavage for 3 days before cisplatin single injection (25 mg/kg). Renal function, apoptosis, inflammation, oxidative stress and p-ERK were measured to evaluate kidney injury. *In vitro*, mouse proximal tubular cells (mPTCs) and human proximal tubule epithelial cell line (HK2) were pretreated with or without IQC (80 μM for mPTCs and 120 μM for HK2) for 2 h and then co-administrated with cisplatin for another 24 h. Apoptosis, inflammation, ROS and p-ERK of cells were also measured. *In vivo*, IQC administration strikingly reduced cisplatin-induced nephrotoxicity as evidenced by the improvement in renal function (serum creatinine and blood urea nitrogen), kidney histology (PAS staining), apoptotic molecules (cleaved caspase-3, caspase-8, Bax and Bcl-2), inﬂammatory cytokines (IL-1β, IL-6, TNF-α, and COX-2), oxidative stress (MDA and total glutathione) and p-ERK. In line with *in vivo* findings, IQC markedly protected against cisplatin-induced cell injury in mPTCs and HK2 cells. Collectively, these findings demonstrated that IQC administration could significantly protect against cisplatin nephrotoxicity possibly through ameliorating apoptosis, inflammation and oxidative stress accompanied by cross talk with p-ERK. Furthermore, IQC may have potential therapeutic uses in the treatment of cisplatin-induced acute kidney injury.

## Introduction

Cisplatin (cis-diamminedichloroplatinum (II), CDDP) is used in many chemotherapy regimens due to its prominent and broad-spectrum antineoplastic characteristics. Because of its distinctive cytotoxic effects, cisplatin gradually causes numerous common and life-threatening adverse reactions, including nephrotoxicity, ototoxicity, hepatotoxicity, gastrointestinal toxicity, neurotoxicity, myelosuppression and so on (Ghosh, 2019). Particularly, cisplatin accumulates in proximal tubular epithelial cells (Xu et al., 2015), thus nephrotoxicity becomes one of the primary and most feared side effects. Hence, the alleviation of nephrotoxicity is a pressing issue. Although not fully understood, there are multi-factorial mechanisms for cisplatin-induced nephrotoxicity including reactive oxidative stress (ROS), reactive nitrogen species, inflammation, apoptosis, fibrogenesis, necrosis and hypoxia (Yao et al., 2007). It is estimated that almost all patients receiving high-dose cisplatin treatment have suffered from nephrotoxicity, including acute kidney injury (AKI), hypomagnesemia and so forth (Manohar and Leung, 2018). Complications from cisplatin therapy attribute to substantial morbidity and mortality, imposing a remarkable economic burden on society worldwide. A vast number of preventive strategies have been constantly explored to manage cisplatin-mediated nephrotoxicity, however, there are few treatment strategies that have been suggested for managing nephrotoxicity induced by cisplatin in clinical practice. So far, hydration with magnesium supplementation and mannitol (Crona et al., 2017) is common, however, its effectiveness is controversial. Cystone was found to exert protective effects (Casanova et al., 2020) in cisplatin-associated nephrotoxicity. Until now, no effective and conclusive therapy is available that can block cisplatin-induced nephrotoxicity. In this context, the uses of antiapoptotic, anti-inflammatory, and antioxidant agents have become primary approaches to develop therapeutic strategies to inhibit or at least reduce cisplatin-induced nephrotoxicity. Intriguingly, combinatorial regimens of herbage have been gaining increasing interest to reduce the side effects of cisplatin. Recently, some reports (Hooshyar et al., 2019; Kim J. W. et al., 2019; Ma et al., 2019; Wahdan et al., 2019) have demonstrated a potential of regimens of herbage in protecting against the cisplatin nephrotoxicity.

IQC (Isoquercitrin), one of the major glycosidic forms of quercetin, is a member of the ﬂavonoids. It is extensively distributed in medicinal herbs, fruits, vegetables, cereals, and beverages (Valentova et al., 2014). IQC has been attracting the attention of various medical and pharmaceutical disciplines in recent years because of its anti-inflammatory, anti-oxidative, anti-apoptotic, and anti-cancer characteristics (Chen et al., 2015; Li et al., 2016; Cao et al., 2017). Research interest in the fields of therapeutic effects of IQC is rising, although most of them have focused on cardiology, orthopedics, tumors and so on (Amado et al., 2014; Huang et al., 2018; Li et al., 2019). A previous study found that IQC was capable of inhibiting cadmium-induced kidney injury via antioxidative effects (Li et al., 2011). However, there are no investigations about the effect of IQC on cisplatin-induced nephrotoxicity.

Therefore, we put forward a hypothesis that IQC will be a suitable candidate for lowering the nephrotoxicity via suppressing cisplatin-induced apoptosis, inflammation, and oxidative stress. This study was designed to determine the effects of IQC on cisplatin-induced nephrotoxicity by evaluating the variation of apoptosis, inflammation, and oxidative stress markers. Our research will provide promising information for a more powerful and pluralistic approach for enhancing chemo-protection against cisplatin-induced kidney injury.

## Materials and Methods

### Reagents

IQC (HY-N0768) and U0126 (HY-12031) were purchased from MedChemExpress (MJ, NJ, United States). Cisplatin was procured from Sigma Aldrich (St. Louis, MO, United States). Periodic Acid-Schiff (PAS) stain kit was ordered from Servicebio (G1008, Wuhan, China). Antibodies against Bax, Bcl-2, Caspase-3, β-actin, GAPDH and Horseradish peroxidase-conjugated Goat Anti-Mouse antibody were obtained from Proteintech Group (Rosemont, IL, United States). MDA assay Kit (S0131), GSH and GSSG Assay Kit (S0053), Reactive Oxygen Species Assay Kit (S0033S) and Horseradish peroxidase-conjugated Goat Anti-Rabbit antibody were provided by Beyotime Biotech (Nantong, China). p44/42 MAPK (ERK1/2) (4695S), Phospho-p44/42 MAPK (p-ERK1/2) (8544S), NF-κB p65 (p65) (8242S) and Phospho-NF-κB p65 (P-p65) (4025S) were obtained from Cell Signaling Technology (Beverly, MA, United States). NGAL antibody is provided by Abcam (Cambridge, MA, United States). Dimethyl sulfoxide (DMSO) was provided from Sigma-Aldrich Chemicals (St. Louis, MO, United States). DAB (SA-HRP) TUNEL Cell Apoptosis Detection Kit (G1507) was from Servicebio (Wuhan, China). All other chemicals used in the study were commercially available and of analytical grade.

### Animals

Adult male C57BL/6J mice (8–10 weeks aged) were purchased from Model Animal Research Center of Nanjing University. Mice were raised in a specific pathogen free (SPF) atmosphere under an alternative cycle of 12 h on/off light. Animals had free access to a standard diet and water ad libitum, and they were acclimated to the facility for 1 week prior to the study. The ethical principles and guidelines were based on the Institutional Animal Care and Use Committee of Nanjing Medical University.

### Establishment of Cisplatin-Induced Nephrotoxicity Model and Treatment Regimen

#### Cisplatin-Induced Nephrotoxicity Following Cisplatin Treatment for 72 h

**FIGURE 1:**
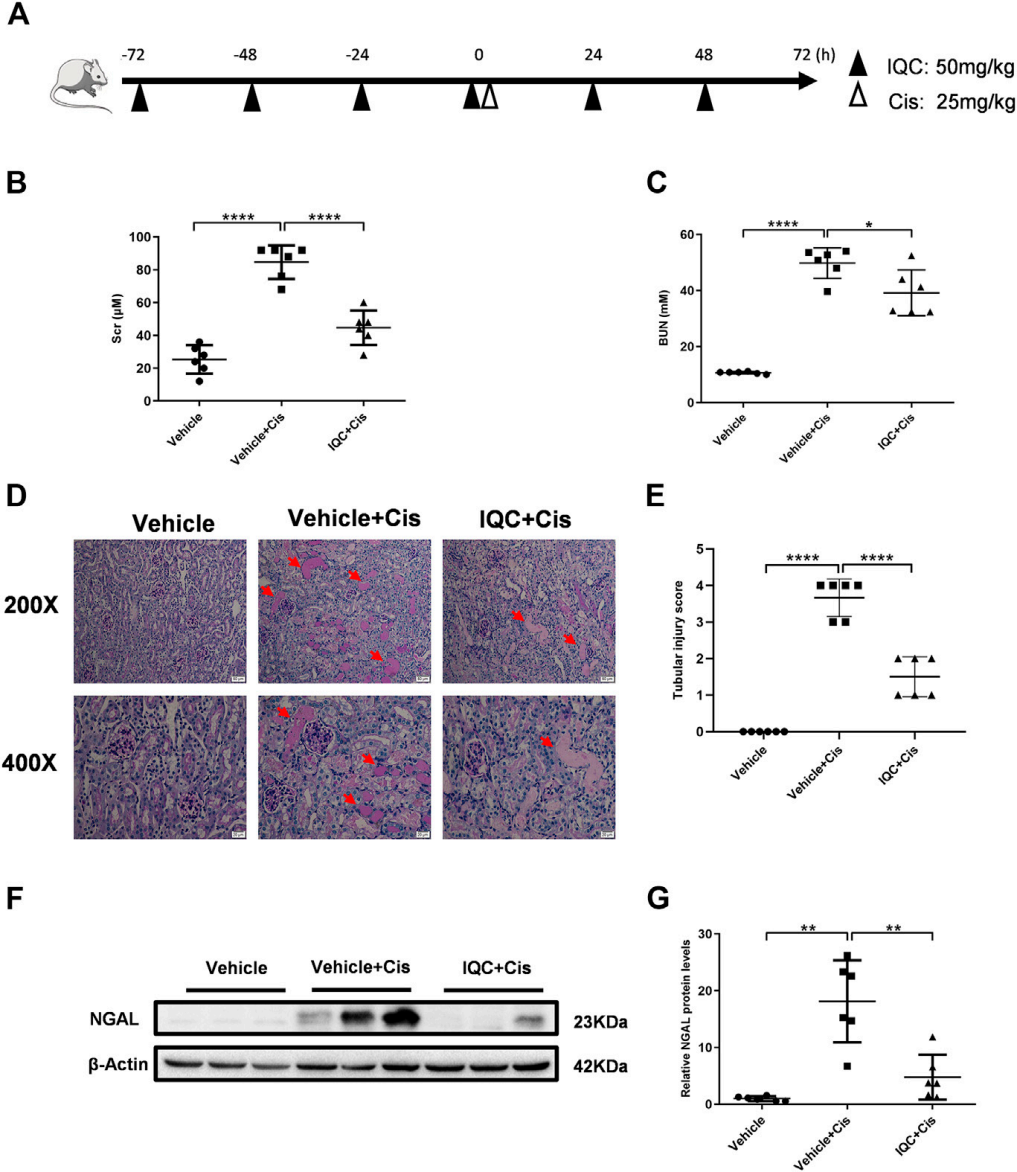
IQC administration protected against cisplatin-induced acute kidney injury. **(A)** Drug administrations and experimental procedures. **(B)** Serum creatinine levels (Scr). **(C)** Serum levels of blood urea nitrogen (BUN). **(D)** Representative images of periodic acid-Schiff (PAS) staining (upper row: magnification ×200, scale bar: 50 μm; lower row: magnification ×400, scale bar: 20 μm) of kidneys. Arrows: tubular cast formation. **(E)** Quantification of tubular injury score of five random fields from each mouse. **(F)** Representative western blots of NGAL protein levels with or without IQC. **(G)** Densitometry analysis of the western blots of NGAL. All the data were expressed as means ± S.D (*n* = 6 mice per group). Cis, Cisplatin; IQC, Isoquercitrin. *p < 0.05, **p < 0.01, ****p < 0.0001.

Eighteen mice were randomly divided into the following three groups (*n* = 6 per group): vehicle (40% PEG300 and 5% Tween-80 in saline) group (Vehicle), cisplatin-alone group (Vehicle + Cis) and cisplatin in combination with IQC group (IQC + Cis). In the experiment, mice were pretreated with 50 mg/kg IQC (IQC + Cis group) or vehicle (Vehicle and Vehicle + Cis group) by gavage once daily for four consecutive days. On the fourth day after 2 h of IQC administration, a single dose of cisplatin or saline (25 mg/kg i.p.) was administered to induce nephrotoxicity. Then IQC treatment was continued by gavage at 24, 48 h after the injection of cisplatin, respectively. After 72 h of cisplatin administration, all the mice were anesthetized with isoflurane and the blood was collected from the inferior caval vein. After that, mice were euthanized by cervical dislocation and the kidney tissues were collected for further analyses. The specific experimental procedure and drug treatment were shown in Figure 1A.

#### Cisplatin-Induced Nephrotoxicity Following Cisplatin Treatment for 24 and 48 h

To further explore whether IQC plays a protective role in the early stage of cisplatin nephrotoxicity, we performed a separate experiment. In this experiment, 36 mice were randomly separated into six groups (*n* = 6 per group): vehicle group (Vehicle), cisplatin-alone group for 24 h [Vehicle + Cis (24 h)], cisplatin-treated group for 48 h [Vehicle + Cis (48 h)], cisplatin plus IQC group for 24 h [IQC + Cis (24 h)] and cisplatin plus IQC group for 48 h [IQC + Cis (48 h)]. In the experiment, mice were pretreated with 50 mg/kg IQC (IQC + Cis group) or vehicle (Vehicle and Vehicle + Cis group) by gavage once daily for four consecutive days. On the fourth day, mice were challenged with cisplatin or saline (25 mg/kg i.p.). Ultimately, mice were sacrificed at 24 and 48 h, respectively, following cisplatin administration (IQC treatment was continued until mice were sacrificed). In addition, 50 mg/kg IQC was given to mice by gavage daily for six consecutive days to evaluate the potential toxicity of IQC on mice.

#### Comparation of the Effect of IQC and U0126 on Cisplatin-Induced Nephrotoxicity

Furthermore, to compare the therapeutic effect of IQC and U0126 in this experimental setting, 24 mice were divided into four groups (*n* = 6 per group): vehicle group (Vehicle), cisplatin-alone group (Vehicle + Cis), cisplatin plus IQC group (IQC + Cis), and cisplatin plus U0126 group (U0126 + Cis). IQC + Cis group was pretreated with 50 mg/kg IQC as mentioned above. As for U0126 + Cis group, mice were administered with U0126 (25 mg/kg i.p.) 1 h before cisplatin challenge. After 72 h of cisplatin administration, mice were sacrificed as described above.

### Cell Culture and Treatment

The mouse renal proximal tubule cells (mPTCs), human proximal tubule epithelial cell line (HK2) and Human Hepatocarcinoma Cells (HepG2) were purchased from American Type Culture Collection (ATCC, Manassas, VA, United States) and cultured in DMEM/F-12 (319-075-CL, Wisent, Canada) or DMEM medium containing 10% fetal bovine serum (FBS) (FBSSA500-S, AusGeneX, Molendinar, Australia). Cells were maintained at 37°C with 5% CO_2_ in a humidified atmosphere and fed with fresh medium once daily. When reached to a 70% confluence, cells were first pretreated with or without IQC (dissolved in sterile DMSO) in DMEM/F-12 or DMEM serum-free medium for 2 h. Then, 5 µg/ml cisplatin for mPTCs, 10 µg/ml cisplatin for HK2, or different concentrations of cisplatin for HepG2 were added for 24 h (dissolved in sterile DMSO). Experiments for renal tubular cells included four groups: control group (control); IQC group (IQC); cisplatin alone group (Cis) and IQC plus cisplatin group (IQC + Cis). For HepG2 experiments, cells were treated by IQC with or without cisplatin.

### Measurements of Serum Biochemical Indicators

Blood samples were collected from the inferior caval vein. After centrifugation at 2,000 g for 20 min, the serum was separated and kept at −80°C until being assayed for BUN (blood urea nitrogen), Scr (serum creatinine), AST (aspartate aminotransferase), ALT (alanine transaminase), LDH (lactate dehydrogenase) and CK-MB (Creatine kinase-MB). The serum’s biochemical parameter levels were evaluated using an automatic biochemical analyzer in the Children’s Hospital of Nanjing Medical University.

### Histological Analysis and Tubular Injury Score

Kidney tissues were rapidly isolated, washed with ice-cold saline and immediately fixed in 4% paraformaldehyde overnight at 4°C. After being embedded in paraffin wax, specimens were cut into 3 μm thickness by rotary microtome (Leica Biosystems, RM2235, Germany). Following drying at 37°C, deparaffinization and rehydration through a series of xylene and alcohol solutions, sections were subjected to PAS stain according to the technical manual (Servicebio, G1008; Wuhan, China) for routine examination under a light microscope. The degree of acute tubular injury was scored by the loss of brush border, tubular dilation, cast formation, and cell lysis. In each kidney tissue, five different randomized areas were assessed and scored based on the following standard: 0, normal histology; 1, 25% of tubules showed pathological damage; 2, 25–50%; 3, 50–75%; 4, >75%.

### TUNEL Assay

Renal tubular cell apoptosis was determined by a DAB (SA-HRP) TUNEL Cell Apoptosis Detection Kit (Servicebio, G1507; Wuhan, China) as instructed by the protocol. TUNEL-positive signals were examined with an Olympus BX51 microscopy (Olympus, Center Valley, PA). Five randomly selected visual fields were checked for the number of apoptotic cells for each sample.

### Measurement of Malondialdehyde, Total Glutathione and Reactive Oxygen Species

To analyze the oxidative stress, we determined the activity of malondialdehyde (MDA) and total glutathione level in kidney tissues via commercially available Lipid peroxidation MDA assay Kit (Beyotime, S0131; Nantong, China), GSH and GSSG Assay Kit (Beyotime, S0053; Nantong, China), ROS Assay Kit (Beyotime, S0033S; Nantong, China) based on the manufacturer’s instructions. Lipid peroxidation was measured by the level of thiobarbituric acid determined as MDA. Both MDA and total glutathione activities were expressed as µmol/g protein. The level of ROS was determined by flow cytometer.

### Cell Counting Kit-8 Assay

Cell viability was analyzed by a CCK-8 assay kit (KeyGen Biotech, Nanjing, China). Briefly, mPTCs, HK2, and HepG2 were seeded and cultured in a 96 well plate. At 60% confluence, cells were treated with IQC of different concentrations in serum free medium for 26 h. In order to detect the effect of IQC on cisplatin-induced cell viability, mPTCs were pretreated with varied doses of IQC for 2 h and then induced with cisplatin (5 µg/ml for mPTCs, 10 µg/ml for HK2 and 20–40 µg/ml for HepG2) for 24 h. After that, 10 μl CCK-8 reagent and 90 μl serum-free medium were added for 2 h at 37°C in 5% CO_2_. The absorbance was detected at 450 nm with a Multiskan FC microplate reader (Thermo Fisher Waltham, MA, United States).

### RNA Extraction and Quantitative Reverse Transcription-Polymerase Chain Reaction

Total RNA of cells or kidney tissues were isolated using TRIzol (TaKaRa, Tokyo, Japan) in accordance with the manufacturer’s instructions. The concentration and purity of total RNA of each sample were quantified by a NanoDrop One spectrophotometer (Thermo Fisher Scientific, Waltham, MA, United States) by measuring the OD_260_ and OD_260_/OD_280_ ratio, respectively. After concentration determination, total RNA was reversely transcribed into cDNA using PrimeScript RT Reagent Kit (TaKaRa, Tokyo, Japan). Real-time PCR amplification was performed using the SYBR Green master mix (Vazyme, Nanjing, China) by means of a QuantStudio 3 Real-time PCR System (Applied Biosystems, Foster City, CA, United States). The expression levels of interleukin-1β (IL-1β), interleukin-6 (IL-6), tumor necrosis factor (TNF-α) and Cyclooxygenase-2 (COX-2) were calculated using the 2^−ΔΔCt^ method with GAPDH as an internal control. The primers sequences are listed in Table 1.

**TABLE 1:** Sequences of the primers for qRT-PCR.

Gene	Primer sequence (5′-3′)
Mouse IL-1β	F: ACT​GTG​AAA​TGC​CAC​CTT​TTG
	R: TGT​TGA​TGT​GCT​GCT​GTG​AG
Mouse IL-6	F:ACAAAGCCAGAGTCCTTCAGAGAG
	R: TTG​GAT​GGT​CTT​GGT​CCT​TAG​CCA
Mouse TNF-α	F: TCC​CCA​AAG​GGA​TGA​GAA​G
	R: CAC​TTG​GTG​GTT​TGC​TAC​GA
Mouse COX-2	F: AGG​ACT​CTG​CTC​ACG​AAG​GA
	R: TGA​CAT​GGA​TTG​GAA​CAG​CA
Mouse GAPDH	F: AGG​TCG​GTG​TGA​ACG​GAT​TTG
	R: TGT​AGA​CCA​TGT​AGT​TGA​GGT​CA

### Western Blotting

The kidney tissues or cells were lysed with radioimmunoprecipitation assay (RIPA) lysis buffer (Beyotime Biotech, Nantong, China) supplemented with 1× protease inhibitor cocktail (Roche, Basel, Switzerland) proportionally 50:1 for 15 min on ice, then samples were centrifuged at the speed of 12,000g at 4°C for 15 min to obtain supernatant. The protein content was measured using the BCA Protein Assay Kit (Beyotime Biotech, Nantong, China). Then equal amounts of protein (30 μg/lane) were separated by sodium dodecyl sulphate polyacrylamide gel electrophoresis, followed by transferring to the polyvinylidene difluoride membranes. After being blocked in 5% milk in Tris-buffered saline with 0.1% Tween-20 (TBST) at room temperature for 1 h, the membranes were then probed with the primary antibodies against cleaved-caspase-3 and caspase-3 (Proteintech; 19677-1-AP, 1:1,000), cleaved-caspase-8 (Cell Signaling Technology; 9496S, 1:1,000), Bax (Proteintech; 50599-2-Ig, 1:1,000), Bcl-2 (Proteintech; 26593-1-AP, 1:1,000), NGAL (abcam; ab63929, 1:1,000), Phospho-p44/42 MAPK (p-ERK1/2) (Cell Signaling Technology; 8544S, 1:1,000), p44/42 MAPK (ERK1/2) (Cell Signaling Technology; 4595S, 1:1,000), phospho-NF-κB p65 (P-p65) (Cell Signaling Technology; 4025S, 1:1,000), NF-κB p65 (p65) (Cell Signaling Technology; 8242S, 1:1,000) at 4°C overnight. After washing with TBST buffer for three times, membranes were incubated at room temperature with horseradish peroxidase-conjugated Goat anti-Rabbit antibody (Beyotime; A0208, 1:2,000) or Horseradish peroxidase-conjugated Goat anti-Mouse antibody (Proteintech; SA00001-1, 1:2,000) for 1 h. All antibodies were diluted in 5% nonfat milk prepared in TBST, and β-actin (Proteintech; 20536-1-AP, 1:2,000) or GAPDH (Proteintech; 60004-1-Ig, 1:5,000) was used as the internal control. Signals of immunoblotted band were detected with the enhanced chemiluminescence detection system (Bio-Rad, Hercules, CA, United States). The intensity of the bands was quantified with ImageJ Analysis software (version 1.51, Rawak Software Inc., Stuttgart, Germany).

### Enzyme Linked Immuno Sorbent Assay

After collection, all the cell supernatant or separated plasma were used to measure concentration of inflammatory factors, TNF-α, and IL-6 by using enzyme-linked immunosorbent assay kits for mouse TNF-α and IL-6 (Dakewe Biotech, No. 1217202, and No. 1210602, Beijing, China) according to the instructions provided by the manufacturers.

### Apoptosis Assay

After gently washing thrice with cold PBS, mPTCs, HK2 and HepG2 cells were lifted from plates by using trypsin free of EDTA. Then, the cells were double-stained with annexinV-FITC and PI with an apoptosis detection kit (BD Biosciences, 556547, San Diego, CA, United States) according to the manufacturer’s instructions. After incubation for 15 min in the dark, the apoptotic cells were examined using a ﬂow cytometer (Beckman Coulter, Bria, CA, United States). Combinations of early and late apoptosis were considered apoptotic and the results were analyzed using CytExpert version 2.0 software (Beckman Coulter, Inc., Bria, CA, United States).

### Statistical Analysis

All data in this study were presented as means ± standard deviation. Statistical analyses were determined by analysis of variance followed by Tukey test for multiple groups. For comparison of two groups, unpaired two-tailed Student’s t-test was performed. Statistical analysis and graphing were performed using GraphPad Prism software (version 8.0.1, San Diego, CA, United States). A value of *p* less than 0.05 was deemed as statistically significant.

## Results

### IQC Treatment Ameliorated Cisplatin-Induced Renal Dysfunction

Our experimental procedure is shown in Figure 1A. Single intraperitoneal injection of cisplatin (25 mg/kg) significantly impaired renal function as assessed by elevated Scr, BUN in contrast to the Vehicle group. Strikingly, administration of 50 mg/kg IQC remarkably attenuated the upregulation of Scr and BUN (Figures 1B,C). Consistent with the improved kidney function, concomitant IQC treatment also exerted a protective effect on cisplatin-treated renal histopathological damage and preserved the renal architecture by staining with PAS. It shows that the Vehicle group exhibited normal, intact kidney morphology, while the Vehicle + Cis group was characterized by severe alterations such as brush border loss, vacuolization, tubular cell necrosis, luminal dilatation, and cast formation. Notably, these deleterious structural lesions were significantly ameliorated after IQC treatment compared to the Vehicle + Cis group (Figure 1D). As for the tubular damage score, cisplatin noticeably aggravated the extent of tubular injury compared to the Vehicle group, whereas IQC significantly decreased the injury score (Figure 1E).To further confirm the effects of IQC against cisplatin-induced renal tubular damage, the protein levels of the specific renal tubular injury marker neutrophil gelatinase associated lipocalin (NGAL) was also assessed. Intriguingly, IQC treatment significantly blunted the large elevation of NGAL levels compared to Vehicle + Cis group (Figures 1F,G).

### IQC Ameliorated Apoptosis in the Kidney of Mice Treated With Cisplatin

**FIGURE 2:**
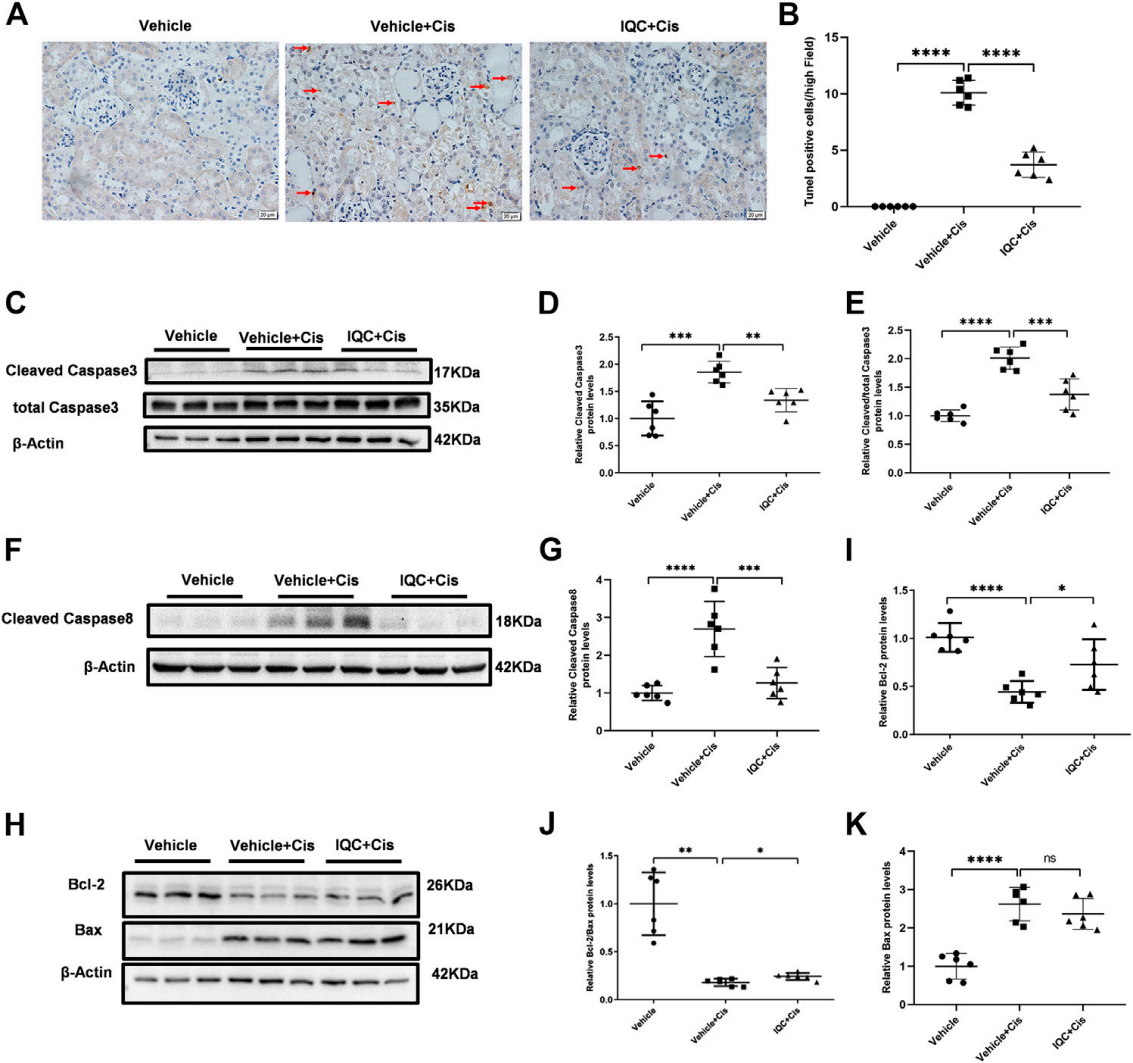
IQC administration suppressed apoptosis in mice treated with cisplatin. **(A)** Representative images of TUNEL staining (magnification ×400, scale bar: 20 μm). Arrows: TUNEL positive cells. **(B)** Quantification of TUNEL positive cells of five random fields from each mouse. **(C)** Western blots of cleaved and total caspase-3 of mice with or without IQC administration. β-Actin was used as the internal control. **(D)** The densitometry analysis of cleaved caspase-3. **(E)** The densitometry analysis of cleaved/total caspase-3. **(F)** Western blots of cleaved caspase-8 of mice with or without IQC administration. β-Actin was used as the internal control. **(G)** The densitometry analysis of cleaved caspase-8. **(H)** Representative immune blots of Bcl-2 and Bax protein levels. β-Actin was used as the loading control. **(I–K)** Qualification of the western blots of Bcl-2 **(I)** Bcl-2/Bax **(J)** and Bax **(K)**. The values were represented as means ± S.D (*n* = 6 mice of each group). *p < 0.05, **p < 0.01, ***p < 0.001, ****p < 0.0001.

Cisplatin-mediated nephrotoxicity is mainly characterized by apoptosis. Therefore, we performed TUNEL staining to evaluate cellular apoptosis in this experimental setting. As shown in Figures 2A,B, the number of TUNEL-positive cells were increased in renal tubules following cisplatin treatment, which was obviously attenuated by IQC treatment. Furthermore, we detected the levels of cleaved caspase-3, cleaved caspase-8, Bax and Bcl-2 by immunoblotting. In agreement with previous studies, cisplatin treatment remarkably enhanced the protein expression of cleaved caspase-3, cleaved caspase-8 and Bax, as well as downregulated Bcl-2 protein expression when compared to the Vehicle group. As expected, IQC treatment largely reversed cleaved caspase-3, cleaved caspase-8 and Bcl-2 expression, displaying a decline in cleaved caspase-3, cleaved caspase-8 and cleaved/total caspase-3 (Figures 2C–G) as well as an incline in Bcl-2 and Bcl-2/Bax (Figures 2H–J). However, we found no significant evidence of IQC in downregulating the level of Bax (Figures 2H,K).

### IQC Blunted Inﬂammatory Response and Oxidative Stress in the Kidney of Mice Treated With Cisplatin

**FIGURE 3:**
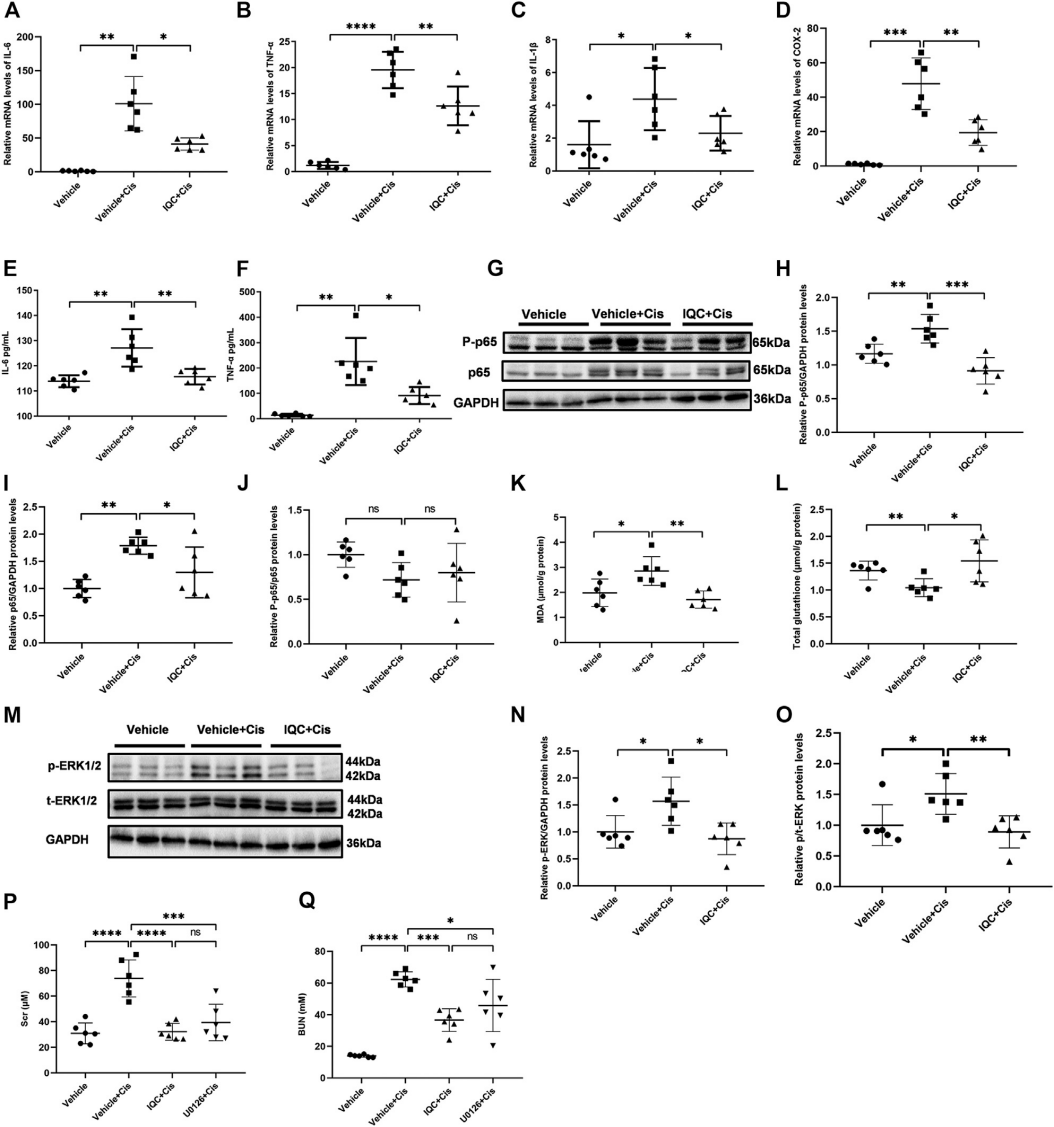
IQC administration suppressed inﬂammatory response and oxidative stress in mice treated with cisplatin. **(A–C)** qRT-PCR was performed to analyze the mRNA levels of renal IL-6 **(A)**, TNF-α **(B)**, IL-1β **(C)** and COX-2 **(D)**. **(E,F)** The protein levels of circulating IL-6 **(E)** and TNF-α **(F)** were analyzed by ELISA. **(G)** Representative immune blots of P-p65 and p65. GAPDH was used as the internal reference. **(H–J)** Densitometry analysis of P-p65 and p65. **(K)** The level of MDA in kidneys of experimental animals. **(L)** The expression of total glutathione in kidneys of mice. **(M)** Representative immune blots of p-ERK and ERK. GAPDH was used as the internal reference. **(N,O)** Densitometry analysis of p-ERK/GAPDH and p-ERK/t-ERK. **(P,Q)** The levels of serum creatinine (Scr) **(P)** and blood urea nitrogen (BUN) **(Q)** when pretreated with IQC or U0126 following cisplatin administration. All the quantitative results were expressed as means ± S.D (n = 6 mice of each group). P-p65, phosphorylated p65; p-ERK, phosphorylated extracellular signal-regulated kinase; MDA, Malondialdehyde. *p < 0.05, **p < 0.01, ***p < 0.001, ****p < 0.0001.

Accumulating evidence demonstrated that excessive inflammation and ROS exerted a role in receiving cisplatin treatment. In line with current studies, IQC pretreatment significantly mitigated the mRNA expression of inﬂammatory factors IL-6, TNF-α, IL-1β, and COX-2 (Figures 3A–D) when compared to the Vehicle + Cis group. Additionally, IQC downregulated IL-6 and TNF-α (Figures 3E,F) at protein secretion levels in serum. Phosphorylated p65 (P-p65) is regarded as primary indicator of inflammation. Hence, we further examined the expression of P-p65 and p65 by immunoblotting. As expected, IQC counteracted the excessive expression of P-p65 and p65 in contrast to the cisplatin group (Figures 3G–J). Besides inflammation, Vehicle + Cis treatment attributed to immoderate oxidative stress. IQC mitigated the pronounced upregulation of MDA and downregulation of total glutathione compared to the Vehicle + Cis group (Figures 3K,L). Furthermore, ERK plays a role in the inflammation process. Thus, we analyzed the level of phosphorylated ERK (p-ERK). As shown in Figures 3M–O, cisplatin treatment markedly increased the expression of p-ERK and depleted under IQC administration. Moreover, previous researches (Jo et al., 2005; Kim et al., 2014) demonstrated that p-ERK inhibitor, U0126, dramatically ameliorated cisplatin-induced AKI. Thus, we further compared the effect of IQC and U0126 on cisplatin-induced AKI and found a comparable protection against renal dysfunction (Figures 3P,Q).

### IQC Protected Against Apoptosis Induced by Cisplatin in mPTCs

**FIGURE 4:**
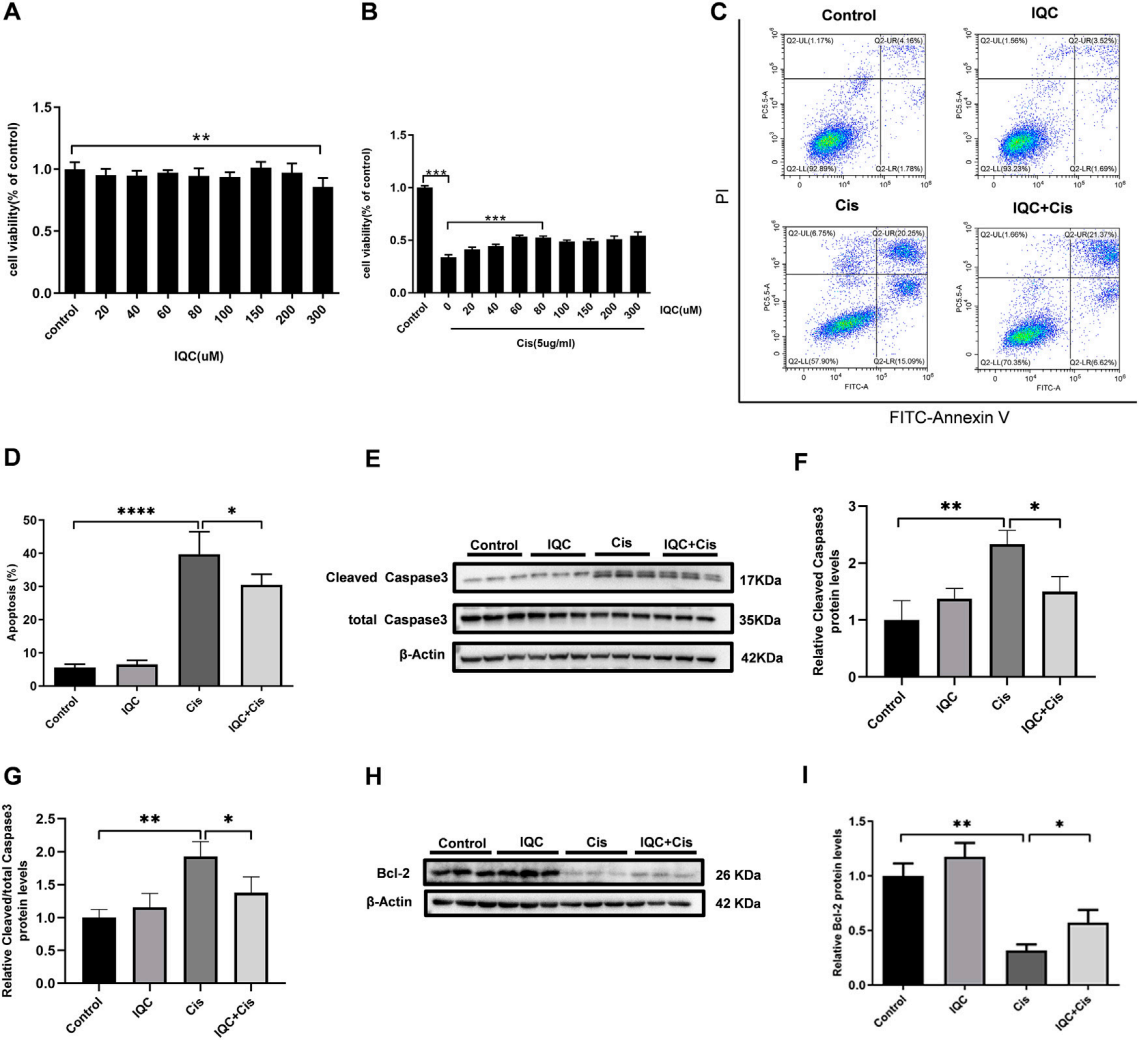
IQC protected mPTCs against apoptosis induced by cisplatin. **(A)** Cell viability of mPTCs treated with varying concentrations of IQC from 20 to 300 µM for 26 h (*n* = 6 per group); **(B)** Cell viability of mPTCs pretreated with different concentrations of IQC followed by 5 µg/ml cisplatin treatment for 24 h (*n* = 6 per group). **(C)** Representative ﬂow cytometry analysis of Annexin V and PI staining. **(D)** Quantification of apoptosis proportion by ﬂowcytometry (*n* = 6 per group). **(E)** Representative western blots of cleaved caspase-3 and total caspase-3 following treatment with cisplatin and/or IQC (80 µM) for 24 h. β-Actin was used as internal control. **(F,G)** Quantitative analysis of the western blots of cleaved caspase-3 **(F)** and cleaved/total caspase-3 **(G)** (*n* = 3 per group). **(H)** The representative western blots of Bcl-2 in mPTCs. β-Actin was used as internal control. **(I)** The qualification of Bcl-2 (n = 3 per group). All the quantitative results were expressed as means ± S.D. *p < 0.05, **p < 0.01, ***p < 0.001, ****p < 0.0001.

CCK-8 was performed to determine the cytotoxic effect of IQC. Cell viability was not significantly affected except exposed to 300 µM IQC alone for 26 h (Figure 4A). A previous study demonstrated approximately half cell viability loss after cisplatin treatment at a dose of 5 µg/ml for 24 h in mPTC (Li et al., 2020). Hence, in our subsequent assays, we used 5 µg/ml cisplatin treatment for 24 h as the cisplatin-induced group which similarly confirmed the remarked cell viability loss in this situation. Additionally, 80 µM IQC treatment significantly attenuated the viability loss in cisplatin group (Figure 4B).

To evaluate the effect of IQC on cisplatin-induced apoptosis in mPTCs, flow cytometry was performed to measure the apoptotic proportion when challenged with cisplatin and IQC in mPTCs. The results showed that IQC largely inhibited apoptosis induced by cisplatin (Figures 4C,D). Consistent with the protective effect of IQC, the levels of cleaved caspase-3 are significantly decreased in IQC + Cis group (Figures 4E,F). In the same way, IQC significantly attenuated the upregulation of cleaved caspase-3/total caspase-3 (Figure 4G). Furthermore, IQC treatment significantly reversed the decrease of Bcl-2 expression (Figures 4H,I).

### IQC Alleviated Inflammation and Oxidative Stress Induced by Cisplatin in mPTCs

**FIGURE 5:**
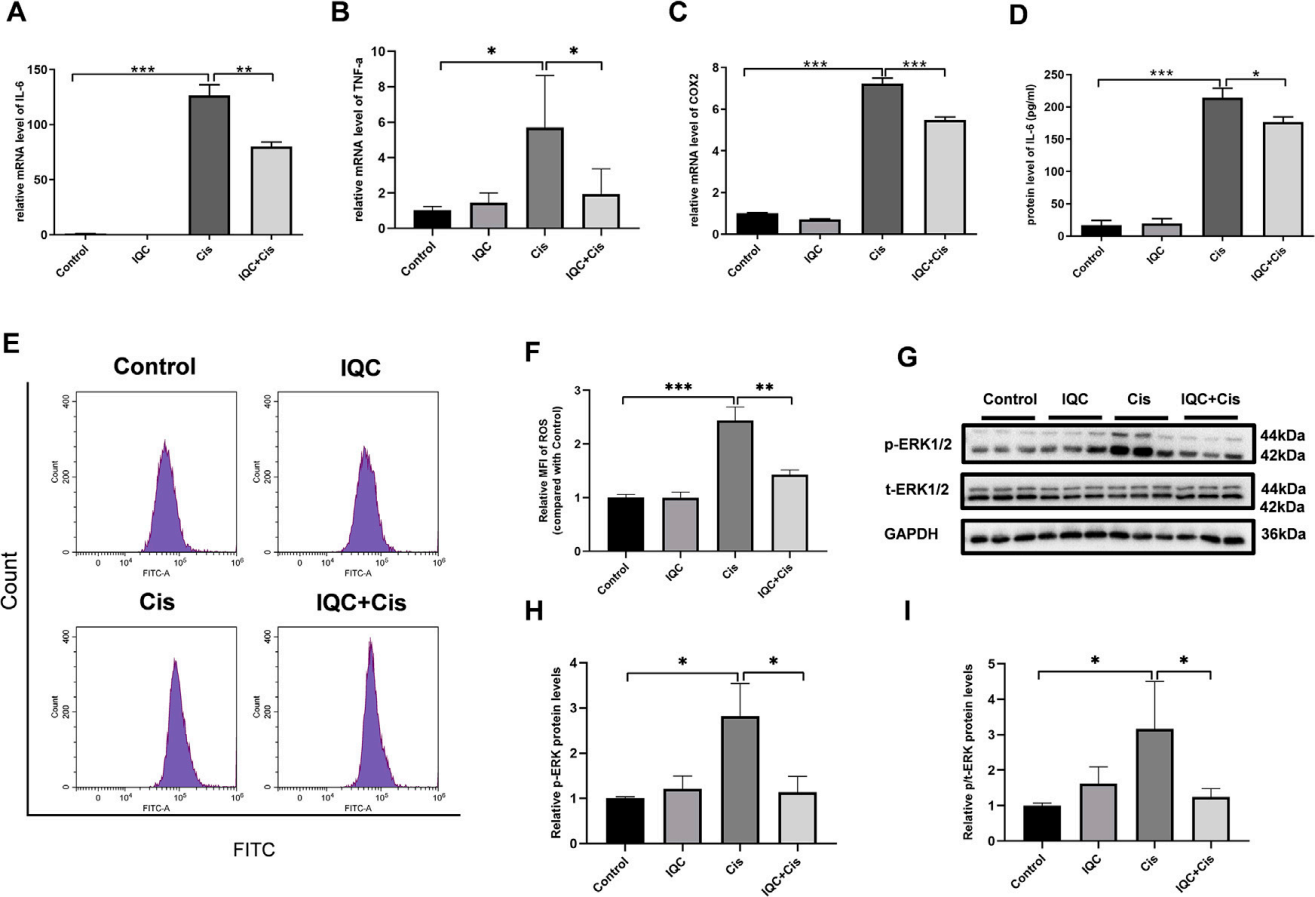
IQC protected mPTCs against inflammation and oxidative stress induced by cisplatin. **(A–C)** The upregulated mRNA expression of IL-6 **(A)**, TNF-α **(B)** and COX-2 **(C)** induced by cisplatin were suppressed by IQC administration. GAPDH was used as internal control (*n* = 6 per group). **(D)** IQC treatment reversed upregulated protein level of IL-6 by ELISA (*n* = 3 per group). **(E)** The representative images of the flow cytometry data from ROS assay in mPTCs. X-axis represents fluorescence intensity, and Y-axis represents cell counts. **(F)** Quantitation of the mean fluorescence intensity (MFI) (*n* = 3 per group). **(G)** Representative immune blots of p-ERK and total-ERK. GAPDH was used as internal control. **(H)** Densitometry analysis of p-ERK/GAPDH (*n* = 3 per group). **(I)** Densitometry analysis of p-ERK/total-ERK (*n* = 3 per group). All the quantitative results were expressed as means ± S.D. *p < 0.05, **p < 0.01, ***p < 0.001.

To determine the effect of IQC on inflammation response *in vitro*, we measured the expression level of IL-6, TNF-α, and COX-2 using qRT-PCR and ELISA in mPTCs. The levels of inﬂammatory cytokines (IL-6, TNF-α, and COX-2) were markedly elevated in the cisplatin group when compared to those in the control group. However, IQC treatment significantly reversed the increase in mRNA expression of IL-6, TNF-α, and COX-2 when compared to the cisplatin group (Figures 5A–C). Similarly, IQC treatment blocked the increase in protein level of IL-6 when compared to the cisplatin group (Figure 5D). Moreover, IQC blocked cisplatin-triggered increase of ROS remarkably (Figures 5E,F). We further evaluated the level of p-ERK *in vitro*, as expected, IQC blocked the marked incline of p-ERK compared with cisplatin group (Figures 5G–I).

### IQC Protected HK2 Cells Against Cisplatin-Induced Injury

**FIGURE 6:**
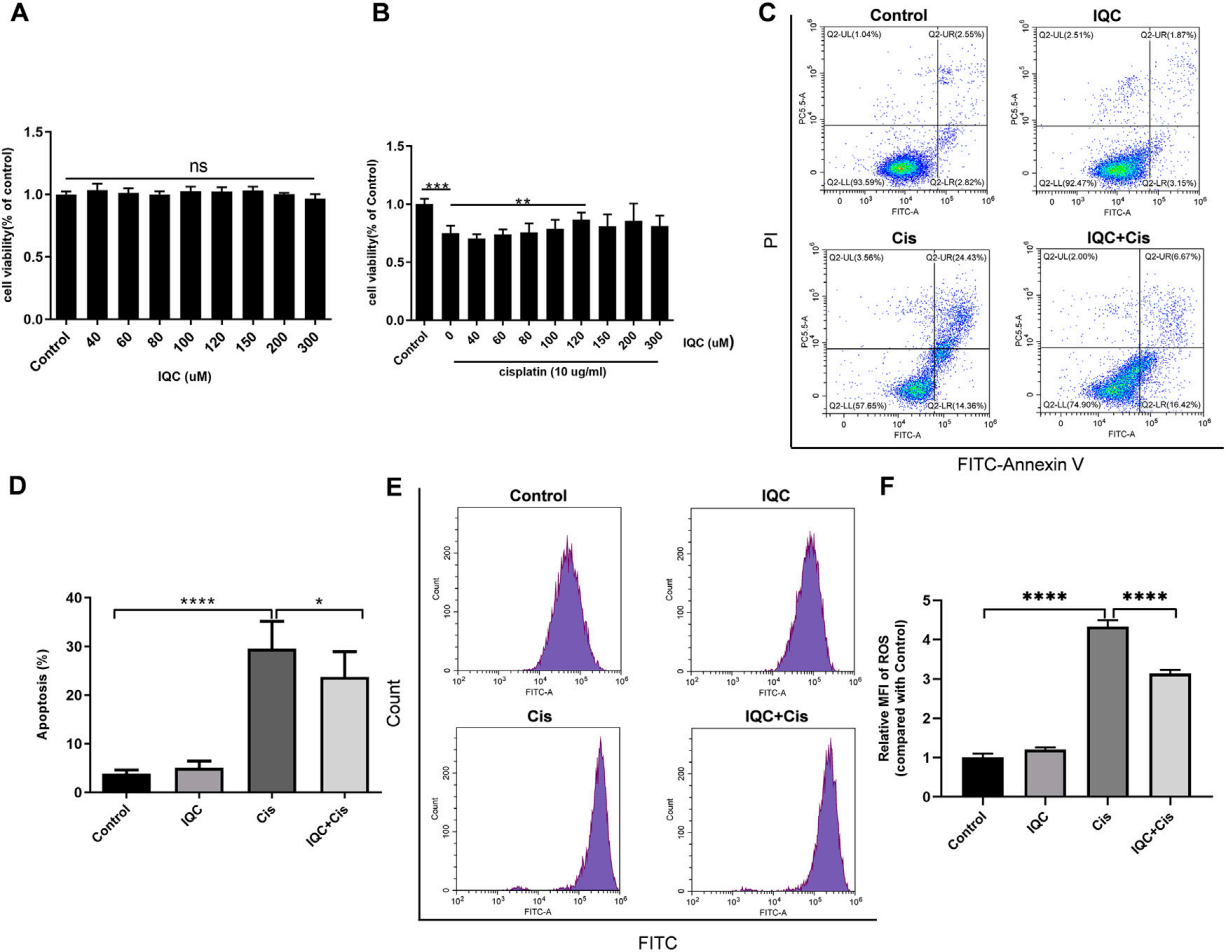
IQC protected against cisplatin-induced apoptosis and oxidative stress in HK2. **(A)** Cell viability treated with different concentrations of IQC from 20 to 300 µM for 26 h. **(B)** Cell viability of HK2 pretreated with different concentrations of IQC followed by 10 µg/ml cisplatin for 24 h. **(C)** Representative ﬂow cytometry analysis of Annexin V and PI staining. **(D)** Quantification of apoptosis proportion by ﬂowcytometry. (*n* = 6 per group). **(E)** The representative images of the flow cytometry data for ROS assay in HK2 cells. X-axis represents fluorescence intensity, and Y-axis represents cell counts. **(F)** Quantitation of the mean fluorescence intensity (MFI) (*n* = 3 per group) The values were represented as mean ± SD. *p < 0.05, **p < 0.01, ***p < 0.001, ****p < 0.0001.

Furthermore, we confirmed the effect of IQC on cisplatin-induced nephrotoxicity in human renal epithelial cells (HK2). Cell viability assay showed IQC at the concentration of 40–300 µM did not impair cell viability in HK2 cells (Figure 6A). Additionally, 120 µM IQC remarkedly conserved cell viability loss under exposure to cisplatin for 24 h (Figure 6B). In agreement with the results of mPTC, IQC strikingly inhibited cisplatin-induced cell apoptosis (Figures 6C,D) and oxidative stress (Figures 6E,F) in HK2 cells.

### Effect of IQC on the Early Stage of Cisplatin-Induced Kidney Injury

**FIGURE 7:**
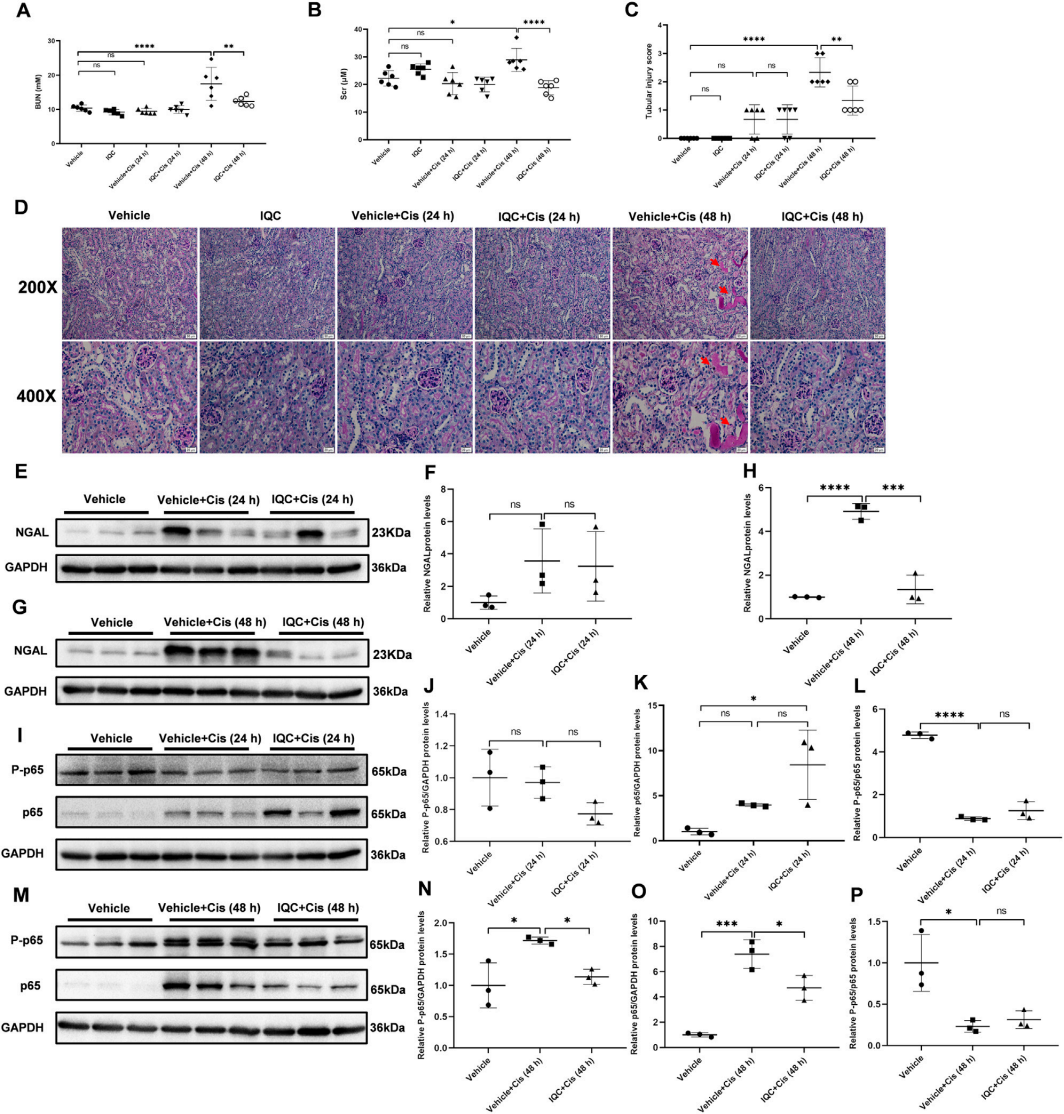
IQC administration protected against cisplatin-induced acute kidney injury at the early stage. **(A)** Serum levels of blood urea nitrogen (BUN). **(B)** Serum creatinine levels (Scr). **(C)** Quantification of tubular injury score of five random fields from each mouse. **(D)** Representative images of periodic acid-Schiff (PAS) staining (upper row: magnification ×200, scale bar: 50 μm; lower row: magnification ×400, scale bar: 20 μm) of kidneys. Arrows: tubular cast formation. **(E,G)** Representative western blots of NGAL protein levels (24 or 48 h). **(F,H)** Densitometry analysis of the western blots of NGAL. **(I,M)** Representative immune blots of P-p65 and p65 at 24, and 48 h following cisplatin challenge, respectively. GAPDH was used as the internal reference. **(J–L,N–P)** Densitometry analysis of P-p65 and p65 at 24 h **(J–L)**, and 48 h **(N–P)** following cisplatin administration. All the data were expressed as means ± S.D (*n* = 3–6 mice per group). *p < 0.05, **p < 0.01, ***p < 0.0001, ****p < 0.0001.

To evaluate the effect of IQC on the early stage of cisplatin nephrotoxicity, mice were sacrificed at 24 and 48 h after cisplatin treatment with or without IQC. As shown in Figures 7A,B, the BUN and Scr levels were significantly enhanced after cisplatin treatment for 48 h, which was abolished by IQC pretreatment. Morphologically, cisplatin treatment resulted in a mild and moderate tubular injury at 24 and 48 h, respectively. As expected, these changes were attenuated by IQC administration at 48 h (Figures 7C,D). Similarly, the regulation of NGAL protein showed a same pattern (Figures 7E–H). We further detected the protein levels of P-p65 and p65 at the time points of 24 and 48 h. As shown by the data, cisplatin injection for 24 h did not increase P-p65 levels even though the total p65 was enhanced. At this time point, IQC had no effect on the expression of P-p65 and total p65 (Figures 7I–L). However, after cisplatin injection for 48 h, we observed an obvious increment of P-p65 and total p65, which was significantly reduced by IQC (Figures7M–P).

### Effects of IQC Treatment on Body Weight, Aminotransferase Enzymes, LDH and CK-MB in Mice With or Without Cisplatin Treatment

**FIGURE 8:**
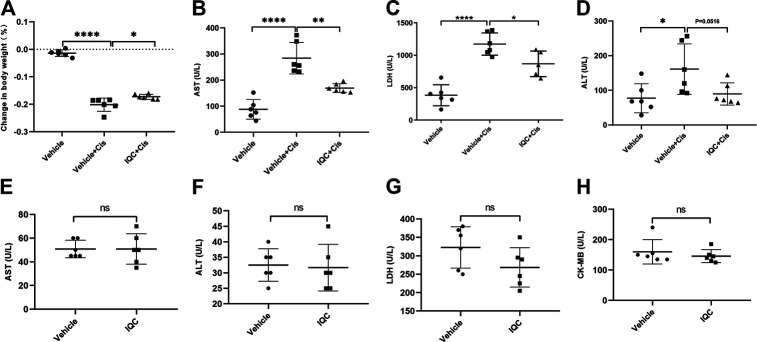
The effect of IQC on body weight, aminotransferase enzymes, lactate dehydrogenase, and CK-MB in mice with or without cisplatin challenge. **(A)** Effect of IQC on cisplatin-induced change in bodyweight and it is calculated according to the formula: (bodyweight at day 7 − bodyweight at day 1)/bodyweight at day 1. **(B–D)** IQC reversed cisplatin-induced increments of AST **(B)**, LDH**(C)** and ALT **(D)** in serum. **(E–H)** IQC alone had no effect on AST **(E)**, ALT **(F)**, LDH **(G)**, and CK-MB **(H)** in serum. All the data were expressed as means ± S.D (*n* = 6 mice of each group). AST, aspartate aminotransferase; ALT, alanine transaminase; LDH, Lactate dehydrogenase. CK-MB, Creatine kinase-MB. *p < 0.05, **p < 0.01, ****p < 0.0001.

During the experiment, we monitored and recorded the bodyweight of each mouse daily. Exposure to cisplatin led to a dramatic loss in bodyweight while IQC administration partially reversed the decline (Figure 8A). To determine the multiple effect of IQC in cisplatin-triggered nephrotoxicity, we also detected other indicators, AST, ALT, and LDH in circulation. Intriguingly, IQC reversed cisplatin-induced increments of AST, ALT and LDH (Figures 8B–D), suggesting a protective role against cisplatin-induced liver injury. In mice without cisplatin treatment, IQC had no effect on the levels of AST, ALT, LDH, and CK-MB (Figures 8E–H).

### Effect of IQC on the Antineoplastic Activity of Cisplatin in HepG2 Cells

**FIGURE 9:**
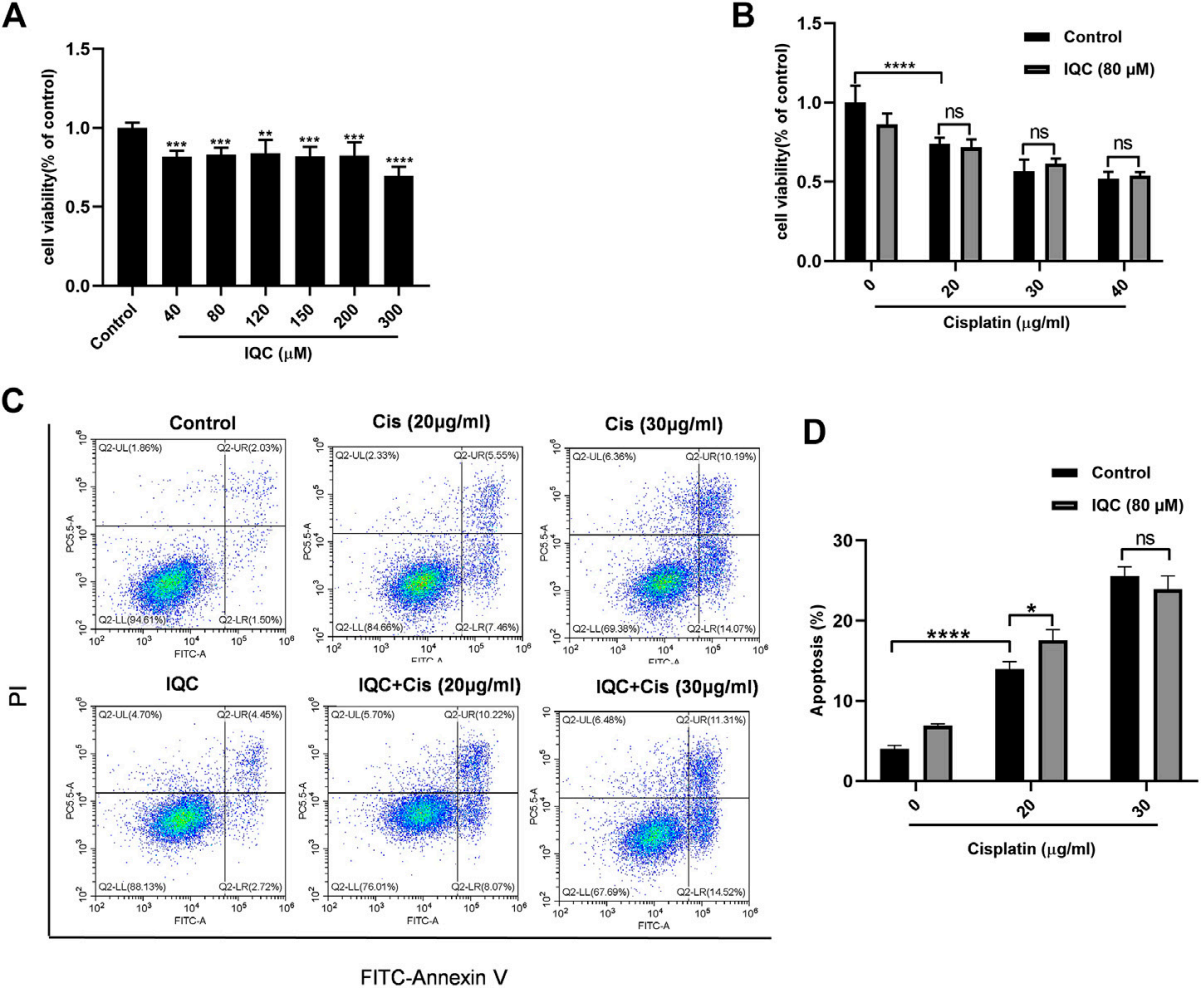
IQC did not affect the anticancer activity of cisplatin in HepG2 cells. **(A)** Cell viability of HepG2 cells treated with different concentrations of IQC from 40 to 300 µM for 24 h (*n* = 6 per group). **(B)** Cell viability of HepG2 cells pretreated with IQC (80 µM) followed by different concentrations of cisplatin (20–40 µg/ml) for 24 h (*n* = 6 per group). **(C)** Representative ﬂow cytometry analysis of Annexin V and PI staining. **(D)** Quantification of apoptosis proportion by ﬂow cytometry. The values were represented as means ± SD (*n* = 3 per group). *p < 0.05, **p < 0.01, ***p < 0.001, ****p < 0.0001.

As shown in Figure 9A, IQC alone dose-dependently inhibited the cell viability of HepG2, suggesting an anticancer potential. However, IQC did not further promote the antineoplastic activity of cisplatin (Figures 9B–D).

## Discussion

Despite the success of cisplatin in cancer, cisplatin-induced nephrotoxicity remains a concern because of the high mortality rate in the cisplatin-associated patients. Therefore, various studies focusing on cisplatin-treated toxicities are being performed. Notably, our current study showed that IQC significantly hampered cisplatin-induced nephrotoxicity *in vivo* and *in vitro* by counteracting unreasonable apoptosis, inflammation, and oxidative stress. According to previous studies, cisplatin treatment induces remarkable kidney malfunction that is characterized by rapid elevation of Scr and BUN. In line with previous studies, our current study confirmed injured kidney function following cisplatin administration. Strikingly, IQC exerted a beneficial role in kidney function displaying as downregulation of Scr and BUN in serum, attenuating the increased expression of ALT, AST and LDH. Additionally, histology examination showed that cisplatin-induced abnormalities in morphology were markedly attenuated by IQC treatment. Furthermore, IQC improved bodyweight loss under cisplatin exposure.

IQC is widely investigated because of its diverse biological activities. First, IQC treatment ameliorated the excessive reactive oxygen species production, and exacerbated the anti-oxygen activity (superoxide dismutase and catalase modulation) *in vivo* and *in vitro* after ischemia/reperfusion (Dai et al., 2018). According to a report, IQC promoted peripheral nerve regeneration following nerve injury through suppress of oxidative stress in mice (Qiu et al., 2019). Furthermore, IQC exerted a protective part in apoptosis. For example, IQC protected from H_2_O_2_-induced apoptosis of EA.hy926 cells, exhibited as downregulation of apoptotic cell proportion and cleaved caspase-3/9 expression as well as upregulation of Mcl-1 (Zhu et al., 2016). Additionally, IQC largely reduced the production of pro-inflammatory cytokines (Li et al., 2016; Huang et al., 2018).

Inflammation is recognized as a critical process in cisplatin-induced kidney injury. Following cisplatin treatment, cytokines such as IL-1β, IL-6, and TNF-α, were markedly expressed, mediating cisplatin-related kidney injury (Faubel et al., 2007; Arjumand et al., 2011). Similarly, *in vivo* and *in vitro*, we confirmed that cisplatin treatment manifested a sharp pro-inﬂammatory reaction, as evidenced by the remarkable rise in the levels of IL-1β, IL-6, TNF-α, and COX-2 compared with the control group. As expected, our results elucidated that IQC normalized cisplatin-induced increase of inflammatory markers. A previous investigation demonstrated that p65 is involved in cisplatin mediated renal toxicity (Zhu et al., 2020). Here, we also observed that IQC decreased the upregulation of both p65 and P-p65. In addition, previous studies (Kim J. Y. et al., 2019; Wang et al., 2020) suggested that levels of anti-oxidant (SOD, CAT and GSH) were depleted, and MDA were increased accordingly under the exposure of cisplatin. Currently, IQC was reported to attenuate oxidative stress to improve high fat diet- and amyloid beta-induced cognitive impairments (Ji et al., 2019). In agreement with these previous results, IQC reversed the upregulation of MDA and downregulation of total glutathione following cisplatin administration which demonstrated the antioxidant effect of IQC in cisplatin-induced nephrotoxicity. These data demonstrated the anti-inflammatory and anti-oxidative stress properties of IQC against cisplatin nephrotoxicity. However, inflammation and oxidative stress always trigger each other. Thus, under current experimental setting, it was hard to define the relationship between the anti-inflammatory and anti-oxidative effects. In the future, we could explore their relationship using a blockade of oxidative stress or inflammation in combination with IQC and cisplatin.

The implication of apoptosis in the pathogenesis of cisplatin-associated nephrotoxicity is well established in the literature (Rana et al., 2001; Badawy et al., 2019). After cisplatin treatment, release of cytochrome C translocated to cytosol (Park et al., 2002), also combination of Apaf-1 and procaspase-9 became an active form, caspase-9 (Hengartner, 2000; Jiang and Wang, 2004). Caspase-9 effectively activates downstream caspases, including caspase 3/6/7 (Hengartner, 2000). Notably, resultant caspase-3 represents the major executioner of apoptosis which is actively involved in apoptosis. Consistent with previous investigations that cisplatin treatment led to cleaved caspase-3 overproduction (Kandemir et al., 2019), in our current study, our results validated that cleaved caspase-3 was upregulated in cisplatin treated groups, however, substantially improved by the pre-treatment with IQC. Particularly, cleaved/total caspase-3 ratio demonstrated a parallel tendency. Moreover, the imbalance between pro-apoptotic protein Bax and anti-apoptotic protein Bcl-2, leads to liberation of cytochrome C into the cytoplasm (El-Kashef and Sharawy, 2018). In previous studies, administration of cisplatin induced an incline in Bax as well as a decline in Bcl-2 (Liu et al., 2014; Tomar et al., 2017). Likewise, in our present research, the expression level of Bax was upregulated in cisplatin treated groups, whereas mildly improved by the pre-treatment with IQC. However, IQC consumption markedly replenished the decline as well as Bcl-2/Bax expression following cisplatin treatment. The marked boost in the protein level of Bcl-2 while slight decrease of Bax clarified that IQC played a more significant anti-apoptosis effect in countering cisplatin-induced nephrotoxicity.

Notably, existing evidence (Potočnjak et al., 2017; Potočnjak et al., 2019) demonstrated that cisplatin attributed to renal expression of ERK1/2, which is associated with apoptosis and inﬂammation. Previous reports (Jo et al., 2005; Kim et al., 2014) showed that inhibition of ERK could protected against kidney injury. Interestingly, in our current study, IQC played a crucial role in cisplatin-induced nephrotoxicity possibly by inhibition of apoptosis, inflammation and oxidative stress accompanied by the downregulation of ERK1/2. Inhibition of ERK signaling with U0126 led to a comparable protection on renal function as compared to IQC therapy, suggesting that the inactivation of ERK signaling might be a possible mechanism of IQC in protecting against cisplatin nephrotoxicity.

Specially, IQC inhibited tumor cell migration and invasion (Xia et al., 2018), and it played a vital protective role in bladder cancer, pancreatic cancer, colon cancer and so on (Amado et al., 2014; Chen et al., 2015; Chen et al., 2016). We also confirmed an antitumor potential of IQC using HepG2 cells. However, we did not observe an additive effect of IQC on the anti-tumor role of cisplatin in this tumor cell line.

The current study clarified the protective role of IQC against cisplatin mediated nephrotoxicity *in vivo* and *in vitro*. Besides, such potent effect is owing to inhibition of apoptosis, inflammation and oxidative stress which is coincided with the decline of p-ERK1/2. Yet, further and in-depth investigations are still required to elucidate detailed molecular mechanism of IQC against cisplatin-induced nephrotoxicity.

## Conclusion

Taken together, our present findings revealed the protective effect of IQC against cisplatin-induced nephrotoxicity *in vivo* and *in vitro* for the first time. Moreover, the mechanisms underlying this effect can be importantly attributed to the inhibition of apoptosis, inflammation and oxidative stress along with the downregulation of p-ERK. Accordingly, it will be a very intriguing point to be studied in the future investigation. Collectively, our findings will supply a new avenue to improve cisplatin-associated nephrotoxicity in clinical application.

## Data Availability Statement

The original contributions presented in the study are included in the article/Supplementary Material, further inquiries can be directed to the corresponding authors.

## Ethics Statement

The animal study was reviewed and approved by Institutional Animal Care and Use Committee of Nanjing Medical University.

## Author Contributions

HC and ZJ designed the study and revised the manuscript. HW, WX, GL, ZP, YL, MW, and QW performed the experiments, analyzed data. YZ contributed to technical advices. Besides, HW and WX wrote the main manuscript text and all authors reviewed the manuscript.

## Funding

This work was supported by grant from Nanjing National Commission on Health and Family Planning (No. YKK18146), and the grant from Nanjing Science and Technology Commission (201823013); grants from the National Natural Science Foundation of China (No. 81873599, 81670647, 82070760 and 82070701); grant of China Postdoctoral Science Foundation (No. 2018M640504); grant of Postdoctoral Fund of Jiangsu Province (No. 2018K255C).

## Conflict of Interest

The authors declare that the research was conducted in the absence of any commercial or financial relationships that could be construed as a potential conflict of interest.
